# Navigating (gendered) social worlds: A qualitative exploration of Canadian young people’s social relationships and mental health

**DOI:** 10.1371/journal.pmen.0000113

**Published:** 2024-11-13

**Authors:** Stephanie Wadge, Valerie Steeves, Kelly A. Pilato, Valerie Michaelson

**Affiliations:** 1 Department of Health Sciences, Brock University, St. Catharines, Ontario, Canada; 2 Department of Criminology, University of Ottawa, Ottawa, Ontario, Canada; University of Santo Tomas College of Science, PHILIPPINES

## Abstract

The purpose of this qualitative study was to explore the gendered ways that youth in Canada are navigating their social relationships, and in turn, how this may be shaping their mental health experiences. Twenty young people between the ages of 11 and 17 (nine self-identified as girls, ten self-identified as boys, and one self-identified as non-binary) were recruited from across Canada and each participated in a virtual individual semi-structured interview. Social relationships were reported as highly important by all participants, and study findings illuminated the strong, persistent, and often implicit ways that these relationships are shaped by gender. This includes the ways that youth describe gender and social relationships influencing experiences and behaviours; how youth manage conflict; and the dissatisfaction that girls express regarding gendered stereotypes, expectations, and norms that they perceive their boy and non-binary peers to experience. Study findings provide context to understanding the gendered disparities that disadvantage all young people. Along with providing new evidence, this paper is a call to action to the adult duty bearers in society to lead changes in how young people are socialized so that they are better equipped to navigate relationships and conflict in positive and healthy ways.

## Introduction

In Canada, one in five people will experience a mental health problem or illness [1]. Similar prevalence levels are reported among Canadian youth [1]. In Canada and globally, a perplexing element of this mental health landscape is a well document gender gap [2–8]. Appearing to emerge during adolescence [8, 9], those who identify as girls consistently report worse mental health than boys across a variety of mental health indicators [8, 10–12]. Research suggests that the increased salience placed on gender socialization and norms during adolescence contributes to these gendered disparities in mental health experiences and outcomes [13]. Yet, the root causes for this gender gap are not well understood. One contributing factor to gendered experiences and disparities in mental health during adolescence may involve social relationships [14–25]. In this current study, we used qualitative methods to ask Canadian young people about their social relationships. Our hope was to provide important insights and contextual knowledge into ways that gendered experiences of navigating social relationships may also shape mental health experiences.

## Background

### Mental health and social relationships

The strength of one’s social relationships and one’s mental health are strongly related [14–21]. Mental health underpins “our individual and collective abilities to…build relationships” [14], and “includes our emotional, psychological, and social well-being” by helping us “relate to others” [15]. At the same time, whether or not one has positive relationships with others also shapes one’s mental health. This bidirectional relationship between social connections and mental health and illness is important across the lifespan [22]. For example, those who are socially connected appear to live longer and happier lives than those who experience social isolation [23]. Further, engagement in supportive social networks also protects against depression [23, 24] and those with a clinical depression diagnosis who perceive their social support as poorer have worse outcomes in terms of symptoms, recovery, and social functioning [25].

### Youth mental health and social relationships

Young people’s social networks expand and develop as they reach adolescence and include their family, peers, and members of their community [26]. When youth feel connected to their social networks, it is positive for their mental health [27].

Parents are an important part of youth social networks. Positive support from parents correlates with a greater likelihood that young people will seek out help or adopt help-seeking behaviours [28]. This is especially the case for younger adolescents who tend to rely more heavily than older adolescents on parental support for help-seeking [29]. When parents are available to their children in both an emotional and physical capacity, youth are also more likely to disclose their mental health difficulties to them and engage in a collaborative help-seeking process [28]. Additionally, when youth report feeling connected to their parents, they also tend to report lower levels of depressive symptoms, suicidal ideation, and non-suicidal self-injury [30, 31].

Since young people spend much of their time in the school environment, teachers also play a critical role in advocating for their students’ mental health needs [32]. For example, teachers are able to engage in promotion (e.g. encouraging inclusivity and positive attitudes toward those with mental illness, promoting healthy lifestyle and exercise, etc.), prevention (e.g. assisting students to manage stress, talking about early warning signs of mental illness, identifying and stopping bullying, etc.), and early intervention (e.g. referring a young person to professional mental health services, asking students about their suicide risk, notifying parents of concerns about their child, etc.) around mental health [33]. When students report a supportive relationship between themselves and their teachers, they also report better mental health [34].

Finally, throughout adolescence, young people begin to place higher importance on their peer connections and are more likely to turn to their friends when they are facing mental health difficulties as they age [35, 36]. Peer support is a protective factor against suicide, depression, anxiety, and stress. It is also positively associated with general mental well-being, self-esteem, and optimism [37, 38]. Various studies indicate that interacting with friends is related to better health outcomes [39, 40]. However, the quality and focus of the friendship is highly important as those who engage with peers who participate in delinquent behaviour are more likely to exhibit mental health difficulties [39]. Overall, when adolescents are dissatisfied with their social contacts or experience poor levels of support, they are more likely to report psychological distress, depression, and anxiety [11].

### Gender and social relationships

Relationships are approached, navigated, and experienced differently by girls and boys [41–44]. Girls appear to place an emphasis on emotional support in their relationships [44–46] and tend to value traits such as loyalty, self-esteem enhancement, and intimacy [47]. Boys on the other hand favour participating in activities with [47] and receiving tangible help and support from their friends [45, 46]. They rarely turn to their social relationships as a coping strategy [48]. Girls are more likely than boys to perceive that their teachers, classmates, and friends support them [49], yet they also report higher levels of stress in their relationships [48]. Exploring these gendered differences in relationships as a potential contributor to mental health disparities may give rise to a more comprehensive understanding of why these disparities exist.

### Gender and adolescent mental health

The prevalence of depressive symptoms [2, 10, 11], anxiety [2, 11, 12], and self-harm [2] is higher among girls compared to boys. Relative to girls, boys have elevated rates of conduct disorder, substance use, interpersonal violence, and completed suicide [50]. They are also less likely to access or engage with professional mental health supports [51–54] and/or possess a strong level of mental health literacy [50]. Additionally, boys tend to experience more self-directed or societal stigma in relation to mental health [55–57]. When compared to their cisgender counterparts, gender diverse young people report experiencing extensive mental health challenges [58, 59], including higher levels of depression, anxiety, suicidality, non-suicidal self-injury, disordered eating, and substance use [59]. While these differences in prevalence among gender diverse youth are becoming well documented, the cause for them is less clear.

### Current study and rationale

In the current study, we hope to shed light on the gendered ways that youth in Canada are experiencing their social relationships. Our rationale is that these findings could provide insight into the social contexts that are maintaining and perpetuating gendered disparities in mental health. As Patalay and Demkowicz (2023) emphasize, “there is little evidence of critical interrogation around *why”* the gender mental health gap exists. Yet in order to close this gap, the *why* needs to “be understood in the context of complex social and societal pressures and power differentials around gender” [2]. By exploring one potential piece of the “why” around this gender gap, it is our hope that our findings can inform health promotion strategies that are attentive to the gendered ways that young people navigate their social worlds.

## Methods

Methodologically, this descriptive qualitative study [60, 61] used semi-structed interviews and thematic analysis [62] to explore and describe the subjective experiences of our participants. Utilizing first-hand descriptions of mental health, social relationships, gender, and the intersections between these topics, allowed for the generation of applied knowledge to be derived directly from the lived experiences of our participants and for our interpretations to remain “closer to the data” [61].

### Sampling and recruitment

Participants were purposively recruited to represent various social identities, with particular attention paid to self-identified gender, between April and November 2021. Conducting interviews with individuals who hold varying and diverse social identities helped to enhance credibility through perspectival triangulation [63]. Due to research restrictions related to the COVID-19 pandemic, youth were initially targeted via emails that were sent to various community youth-based organizations (i.e. community youth centres, a soccer club, and a youth yoga studio). Since most of these organizations were running at a limited capacity during the pandemic, we also created a digital poster that was promoted via a social media campaign on both Twitter and Instagram. Digital posters were also posted through Brock University’s Lifespan Institute. Participants representing three Canadian provinces (Ontario, British Columbia, and Quebec) requested to participate in this project. If interested participants did not live in Canada, did not speak English, or were not between the ages of 11 and 17, they were excluded from the study.

### Generating data

Participants completed a virtual semi-structured interview via Zoom with one interviewer (K.A.P). Prior to beginning the interview, a guardian of the participant provided verbal consent and the youth participant provided verbal assent. The interviews lasted between 45 and 60 minutes each. Participants were asked to reflect on, discuss, and provide stories about their own everyday lived experiences related to gender and mental health, which allowed them to guide the conversation in ways that were meaningful to them. Co-creating data with our participants in this manner enhanced data dependability. In keeping with our methodological approach, the specific questions that were asked evolved and became more focused as data collection progressed. Following the interview, each participant was given a $30 e-gift card to thank them for their time. Each audio recording was professionally transcribed verbatim for subsequent analysis. Data generation was complete once we reached data sufficiency, which was determined when we had adequate interpretive evidence to answer our research question [64].

### Analysis

The main set of 20 transcripts was used to form a meta transcript of all project data. All data related to social interactions and relationships was then compiled into a sub data set for this specific analysis. We completed a first level of line-by-line coding on early transcripts, looking for initial codes in the data that we then discussed. As we engaged in this process, we were attentive to the intersection of different social locations (i.e. participant demographic characteristics such as gender). The first round of open coding focused on understanding and comparing what types of relationships the young people were discussing and how they experienced these relationships in their daily lives. As themes and categories were identified, a gendered analysis allowed for the similarities and contradictions within and between gender identities to be highlighted. In the second phase of coding, a conceptual level of analysis took place and the ways in which young people navigate various relationships were explored. This was also approached from a gendered lens in order to look for similarities, contradictions, inconsistencies, and patterns that were beginning to emerge. In the final phase of analysis, we moved to interpretation. Here we drew from the academic literature, direct participant quotes, and analytic memos to understand how young people appear to be navigating their relationships in gendered ways, and the implications that this may have for mental health.

### Establishing rigor and validity

Throughout all stages of the research we (S.W., K.A.P., V.S., and V.M.) were reflexive about our own social locations and how they intersect with our own lived experiences to influence this work. Our diverse disciplinary backgrounds include academic training in sociology, health policy, law, and community health. As cisgender women of different ages and at different career stages, we had open and reflexive discussions about the different socio-cultural norms and expectations that we have experienced in our own lives. Rigor and validity were further enhanced by having many critical conversations within the research team. We used these conversations to assess what was going on in the data and to familiarize ourselves with the stories that were being told to see how these fit into the broader literature base. The detailed field notes that were taken initially by the interviewer (K.A.P.) were also referenced regularly. As a form of accountability, one author (S.W.) met with an established youth advisory panel. Her purpose was to discuss our analysis process to gain insights about what, from the experiences of the youth advisory members, we should be sensitive to in the data. This discussion added an element of trustworthiness [63] to our investigation. Finally, all researchers engaged in reflexivity throughout the research process and a detailed audit trail of decisions, discussions, and questions was maintained throughout the analysis and write-up of this manuscript. All of these strategies enhanced the validity of our data and contributed to the trustworthiness and integrity of our study.

### Ethical considerations

Ethical approval was received from Brock University (19–099), Queen’s University (EPID-688-19), and the University of Ottawa (S-12-19-5329). To protect the privacy of each participant, any identifying information was removed from the transcripts prior to analysis by the research team and each participant’s name was replaced with a pseudonym. Additionally, any personal information that was used throughout the recruitment process was stored on a password protected computer, separate from the audio files and transcripts.

## Results

### Characteristics of study population

We interviewed twenty young people between the ages of 11 and 17 years. Nine participants self-identified as girls, 10 participants self-identified as boys, and one participant self-identified as non-binary gender. Geographically, one participant was from Quebec, one was from British Columbia, and 18 were from Ontario. We asked participants to self-identify their ethnocultural background on a pre-study demographics form. Thirteen of the participants self-identified as being “Caucasian” or “White” and three participants self-identified as being “racialized.” The other four participants self-identified in ways that did not describe race (such as “Catholic Canadian” or “French Canadian”). Throughout the text the participant’s gender and age are indicated in parenthesis (G: girl, B: boy, NB: non-binary).

### Young people’s experiences navigating social relationships

Findings from our study demonstrate the strong, persistent, and often implicit ways that gender shapes social relationships. This involves how youth describe the ways that gender and social relationships interact to shape behaviours and experiences (theme 1), the ways that youth navigate the conflicts that they experience in their social relationships (theme 2), and the ways that girls describe their dissatisfaction with gender stereotypes, particularly those that are directed at boys and gender diverse youth (theme 3). All themes are described in detail in the following sections.

### Theme 1: Gender and social relationships interact to influence behaviours and experiences

The youth in this study spoke about the many different types of relationships they maintain, including those with their friends, family, and community members such as coaches and teachers. Through our analysis, it became apparent that gender norms, expectations, and social relationships interact with and reinforce one another.

#### Subtheme 1.1: Gender and friendship are interrelated

The young people in this study spoke at great lengths about their friends and the importance they hold in their lives in general and to their mental health in particular. Many of the young people described what makes a good friend. Traits that they value included being “nice”, “energetic”, “honest”, and “loyal.” Yet, the ways that participants described the characteristics of a good friend was gendered. For example, many of the boys in this study placed high importance on wanting their friends to enjoy the same activities and hobbies that they do. Aidan (14B) said:

my friends, they are just people who have the same personality as me, and they want to do the same things as me. So that is helpful because I want to do those things and it’s fun to do them with other people instead of just by yourself.

Connor (16B) echoed this sentiment and said that the qualities that make good friends are that they are “very energetic and they want to do lots of things, because that is what I want to do. Most of my friends are pretty much doing the same thing that I am doing.” Kevin (12B) stated his friends were all “similar” and they “all love sports and having fun and joking around” and he gets along especially well with his “older boy cousins” because they “have lots of fun swimming, playing sports, and hanging outside.”

The girls in this study also spoke about similarities between themselves and their friends. Although they talked about the importance of enjoying the same activities, when compared to the boys, the girls were more focused on the importance of sharing similar goals. Haley (16G) explained that her “closer friends are more similar to me. And not even similar interests, but similar goals.” Alison (14G) had similar feelings and said that “usually friends have the same goals and where they want to be and what they want to do.” Additionally, the girls in this study reported that having their friends express care and support was essential. Alison (14G), Haley (16G), Nicole (13G), and Faith (13G) all expressed that friends were people that they could “count on.” Alison (14G) said that friends “should always be there for you and always be nice to each other” and Haley (16G) wanted her friend to be “someone who has your back…someone that supports you.” Faith (13G) said that friends “make you feel good about yourself and don’t make you feel bad about yourself” and for Nicole (13G) it was important that “they care about you.” While both the boys and girls in this study described the desire for their friends to be similar to them, the manifestation of these similarities reinforced gendered patterns with boys seeking activity-based likeness and girls pursuing friendships in which their goals and identity are supported.

In addition to describing the qualities they look for in their friendships, all participants also described the pressure to look good in front of their friends in a variety of gendered ways. While the youth described that their friends were often the people they turned to in difficult situations, a common point of discussion across gender identities was that friends are often the ones who place expectations and pressure on young people to behave and perform in a certain way.

Nicholas (17B), who said he “wants to look good in front of his friends”, perceived pressure especially from his boy peers. For example, he said:

There is a math contest at our school…. And a lot of the times they preselect boys instead of girls. So, there is a majority of boys. And it’s not necessarily their fault, they pick the boys and girls with the best grades. And a lot of the time it is the boys, so I sort of feel pressure from the other boys that I need to perform well to make it seem like we are better.

While Nicholas described the pressure he felt to be academically “better” than the girls, Elizabeth (13G) described multiple instances in which social media perpetuated gender expectations for girls to replicate the “perfect” appearance, identity, and behaviour. She explained that “everyone on TikTok has the exact same feel of how they should appear.” Elizabeth (13G) said that for her personally:

If it wasn’t for TikTok, I probably wouldn’t be like me, I guess. I probably wouldn’t have some of the clothes that I wear now if it wasn’t for TikTok. So, I think the expectations get bigger and bigger every day.

These growing expectations surrounding appearances rapidly evolve, yet they are propelled by the constant requirement for alignment and sameness. Elizabeth explained that:

People say there is a perfect girl. I mean people are like, oh yeah you have to wear perfect bikinis and have to wear skirts and skinny skirts and all this stuff. You have to make this ice-cap. Oh my gosh you have to do this; this is a trend. You have to do this. Or else you are not going to be the actual expectation of the girl that is supposed to be out there in 2021, I guess. *laughs* I mean all my friends feel the exact same. I have talked to my friends about that. It is weird because we all look the exact same because everything is this trend basically. I can’t even explain it. We all wear the exact same clothes and stuff. It’s weird.

Alex (14NB) described stereotypes that exist for non-binary individuals and the ways that their gender non-conforming peers expect them to behave and present themselves in order to be “considered non-binary.” Alex (14NB) explained: “The majority of my friends are non-binary or fall onto the gender spectrum somewhere. They would say some things and I would be like, well I don’t think that is completely accurate.” For example, according to Alex’s peers and social media, many non-binary folks listen to certain music, enjoy certain hobbies, and “should be androgenous.” This led Alex to feel like they needed to change their behaviour in order to ‘accurately’ display their gender.

People would say things online and that would make me think and wonder, I guess I need to listen to this certain band…and that made me want to change certain interests and hobbies that I have and be like, I guess I have to be this now.

It appears that Alex experienced pressure from the online world to portray themselves in a particular gendered way (similar to the online pressures that the girls in this study reported) while simultaneously feeling the need to align their hobbies and interests with those of their peers (an experience that the boys in this study emphasized). Alex stated that “pushing non-binary into a binary” could be “hurtful to a lot of non-binary people.”

What was particularly salient throughout this section are patterns that seem to appear based on gender socialization and gender norms within friendships (i.e. boys pursuing friendships in which they can participate in the same hobbies, girls seeking out friendships in which their identity and goals are shored up by one another, boys feeling pressure from their same gender peers to outperform girls, girls feeling pressure from online friendships to achieve an ideal (and inherently unachievable) lifestyle, non-binary youth feeling pressure from their peers to express certain interests or appearances, etc.). The pervasiveness of gendered stereotypes, patterns, and experiences are such that they impact the friendships of girls, boys, and non-binary youth.

#### Sub-theme 1.2: Gendered expectations occur in family

All participants often brought up that talking to their parents was their first line of defence against poor mental health. At the same time, participants also reported that their parent’s expectations frequently revolved around traditional gender norms and stereotypes.

Nathan (15B) felt expectations from his father and spoke to the intersection of gender, birth order, and cultural pride. He stated:

My dad is manly…I am expected to do more because I was the first-born son, so I have to do everything…My dad’s first name is my middle name because that was something they did. And I am just older than my brother and so he trusts me more and he thinks we are close. So, I do more…It is annoying, but I feel like I am more important than the other ones [siblings].

Cole (11B) felt pressure from his father around academics. He described a scenario where he was asking his father for help with homework and it left him feeling “disappointed” because instead of being provided with the assistance he needed, his father responded by saying “you should know this.”

While the boys described expectations from their parents to “do more”, some of the girls described the burden they carry within their family systems. Sierra (16G) expressed feeling a burden to support her parents. She explained that:

Even with my parents, I am like, oh they are so stressed. They have so much on their mind…And so, when I’m stressed, I am like, I can fix this myself. I am good. I can do this. It has worked out so far.

When probed to understand this further, Sierra explained that:

my parents are great. They are accepting and I am really comfortable with them. It’s more the burdening part. There are four of us as well and I am the oldest and so I feel like I have a duty to take care of myself sometimes, especially when my siblings have more boundaries to go past then I do. I feel like, you know what I can do this myself.

Sierra’s perception of being a burden and having the duty to take care of herself emphasize the gendered expectations that can occur within the family dynamic.

#### Sub-theme 1.3: Adults in the community reinforce gender expectations and norms

Participants also spoke about the expectations that were placed on them by other adults in their lives, including their coaches and teachers. Zachary (11B) felt the pressure of needing to perform better athletically than his girl peers: “With running though boys are usually faster than girls. So, if there is a grade 11 girl that passes me…my coach will be like, you should have pushed more.” For Zachary, the gendered expectation surrounding his athletic performance was explicit. In a similar way to how Sierra expressed that she didn’t want to burden her parents, Elizabeth (13G) expressed that she did not want to burden her teacher at school. She described how she was struggling in class but didn’t want to “bother” her teacher because there were “28 kids” in her class and the only way to receive help was through virtual breakout rooms (this was because of COVID-19 related online learning public health requirements in Canada.) For Elizabeth, the gendered expectation around caring for someone else’s emotional burdens appeared to be implicit.

### Theme 2: The way participants navigate conflict is gendered

Participants described various scenarios where they were in conflict with people who they saw on a daily basis such as family members, friends, and peers, or those that they interacted with less frequently such as members of their church community or customers at their job. Although all young people experienced some type of conflict in their relationships, how they navigated this conflict appeared to be different depending on their gender identity.

#### Subtheme 2.1: Boys navigate conflict by moving away or disengaging

Boys who participated in the study described navigating conflict by moving away or disengaging. They often used words such as “not being in the way”; “moving away”; “leaving”; and “isolating” from the “energy of the environment” or “doing something to forget about it” to describe how they navigated conflict. Illustratively, many of the boys in this study described conflict with their siblings. Nathan (15B) said that his siblings “talk a lot and they are annoying” and Austin (14B) said that his siblings initiate conflict by blaming him for things he didn’t do, which in turn causes arguments between him and his mother. Nicholas (17B) also described conflict with his mother. “My mom startles really easily. And so, we try to not be in her way…because if she startles there is immediate conflict.” Finally, Zachary (11B) described experiencing “issues” with his friends. Each of these boys explained how they handled the conflict they had described by removing themselves from the situation and finding another activity to do that would distract them. When Nathan (13B) was mad at his siblings, he “would just leave. He just moves away” and for Zachary (11B) he would “go up to [his] room and play video games or read or do something to forget about it.” Austin (14B) found that getting outdoors and “going for a bike ride” helped him handle his frustration toward his mom. For Nicholas (17B), it was more than just escaping the physical environment and the person he was in conflict with; he also needed to find space away from the “energy” of the conflict. He stated that:

I go back to my room because I tend to absorb the energy of the environment and I don’t like to be mad. So, I will isolate myself more instead of confronting the conflict…I watch YouTube videos or draw or listen to music…it calms me.

#### Subtheme 2.2: Girls navigate conflict through sociality and by “looking at the other side”

When girls in the study described how they navigated conflict, they used language around sociality and relationships. Many of the girls described being troubled when they experienced conflict in their relationships. They often told lengthy stories about miscommunication with friends, misunderstandings with family, and the level of “stress” they felt when they were in conflict. Some of the girls reported that they initially navigated conflict by denying it was happening. For example, when Sierra (16G) was describing a conflict within her friend group she stated: “I didn’t see it because I was in denial. I was like, no they wouldn’t do that, what are you talking about? We have been friends for so long.” Similarly, Faith (13G), who was in conflict with one of her close friends at the time of this interview, explained that she “kind of just pretends that it’s not happening and that it’s fine.” However, for both girls, this strategy was only an initial step in navigating the conflict. Both girls described also confiding in other people about the conflicts they were experiencing. Sierra (16G) said that on numerous occasions she “went to another group of friends for a bit…to try and give the people space.” She explained that with her other friends they would “talk about it and try to understand each other…because then it shows you are not alone in the situation.” Similarly, Faith (13G) said that she knew that she had “other friends” that she could spend time with. Elizabeth (13G) described how she would often distract herself by going on her phone when she fought with her brother, but it was to interact with others through social media. “Talking to my friends on Snapchat makes me happier.” Haley (16G), who described conflicts with customers at her job, explained that working with “one of my friends” and “just being around her” made the stressful conflict better. Interestingly, later in our conversation about conflict with her friends, Sierra contradicted herself and stated “I didn’t really want to share what was going on. I mean I did, but I didn’t go into depth about it because I didn’t want to burden other people about what was going on in my life.” While Sierra didn’t want to feel alone, she also was sensitive to relationship dynamics and didn’t want to be a burden, which is similar to the way she responded to her parents as seen in subtheme 1.2.

While many of the girls described the distress that conflict caused them, some of the older girls simultaneously expressed empathy for those with whom they were in conflict. Take Sierra’s (16G) experience for example. When she discussed the conflicts she experienced with her friend group, she stated that she was “always trying to see both sides of the argument…I tried to look at the other side and see where they were coming from.” While seeing her friend’s perspective allowed her to step back from the conflict at hand, it also introduced another layer of stress. “That got really stressful because, yeah, me being the one who tries to resolve all of it myself, I feel like if things are not going good then it is my fault.” When Haley (16G) dealt with challenging customers at her pharmacy job, she also expressed empathy for their situation- “I get that people are upset because they are sick, or someone is sick in their family.” She went on to describe how in some circumstances she experienced frustration at work because some of the older patients insisted on talking to her co-workers who are men instead of listening to her. Despite her frustration, she realized that “they grew up like that. So, I guess I am kind of understanding about it.”

#### Subtheme 2.3: Navigating conflict through self-reflection & self-development: a non-binary experience

Alex (14NB) described experiences of being in conflict with their peers at school, saying “The main reason why I got bullied because when I was younger, I had a weird personality. I agree that I was a pretty weird kid. I also had interests that people said, ‘that is weird.’” In order to navigate this challenging time, they would “cope just by doing art. That is what reflects my emotions, doing vent art. That helped a lot.” The bullying also extended to Alex’s (14NB) appearance and in order to combat this they “would just try different self-esteem exercises. I would find them online or I would talk to my parents about it.” In contradiction to the boys who would isolate and the girls who would deny conflict was occurring, Alex (14NB) reflected on their experiences with conflict. They said:

the main thing is talking to people about it or having time to reflect on things. And of course, if something really bad happens then I don’t want to pretend like it never happened. I will be like, well if I pretend that it didn’t happen then I don’t think I will go through development. And I won’t actually recover. So, I will need to think about it for a bit.

### Theme 3: Some girls described dissatisfaction with gendered stereotypes, expectations and norms that boys and non-binary youth experience

Some of the girls in this study described dissatisfaction with—and resistance to—the gendered stereotypes and norms that their peers encounter in their daily lives. They also expressed recognition of the ways that their peers’ gendered experiences may differ from their own. For example, Sierra (16G) explained that while she identified as cisgender, she recognized that there might be scenarios in which her gender diverse peers could be uncomfortable. She said, “there are times when in general people are like, girls go to that side and guys go to that side. And I am like, what if there are people who don’t identify as either? Or switch?” Ashley and Haley identified the stereotypical emotional repression boys are subjected to. Ashley (13G) described that:

there is a big main thing where some people think that boys should be this big tough muscular man or person that doesn’t cry that much or barely gets emotional. But reality is, boys are human like us, and they have feelings and emotions. So, some people think they should be big and tough and don’t get emotional, but they still have feelings and emotions.

Haley (16G) echoed these thoughts as well.

Even for boys, for example not crying or not being emotional is a thing, which is sad. I feel like we are starting to get rid of that thought. But still, it’s like, oh you have to tough and stuff like that for boys. And when they show an emotional side it’s like, oh my god they are not robots.

A noteworthy feature of our data was that while some of the girls described their dissatisfaction with gendered norms and expectations that they perceived boys and non-binary people experience, none of the boys or expressed this level of recognition, reflexivity, or resistance to gender norms and stereotypes that the girls or non-binary youth may be experiencing.

Nicholas (17B) was an anomaly among the boy participants in that he articulated how he was attentive to the gender stereotypes that influenced his own understanding of gender. For instance, Nicholas (17B) explained that:

There is the classic stereotype of the strong man and super manly like ‘errr’. But as being a gay person, I am not really attached to that stereotype because of the other stereotype that gay boys are flamboyant and girly. So, I try to be in the middle. I don’t identify as girly, more manly…Stereotypically, for example I don’t enjoy watching sports and I like painting and stuff like that. I also wrote for the student journal, and it was almost only girls. So, I do girly things that are not considered manly.

It may be that due to certain aspects of his social position (i.e., sexual orientation), Nicholas displayed a level of gendered resistance in the way that his portrayal of his self-identified gender did not align with what he deemed the dominant depiction of a boy.

## Discussion

The goal of this study was to explore the gendered ways that Canadian young people navigate social relationships. Recognizing the strong connection between social relationships and mental health, our overall hope was to provide insight into the social contexts that shape gendered disparities in mental health. Our main finding was that while social relationships were described as similarly important for all participants, many of the way that the boys, girls, and gender diverse youth navigate the relationships in their lives were highly gendered. In turn, these relationships strongly impact their mental health. This overarching finding suggests that the perceived strong advances in gender equality in Canada may be naively optimistic, and indeed, that advances towards gender equality have not gone nearly far enough. Healthcare providers, parents, teachers, and others who care about, and for, children must become much more sensitive to the gendered ways that young people experience mental health challenges, and further, be more sensitive to the ways that gendered stereotypes exacerbate mental health disparities or isolate young people. Everyone who cares for children needs to work together to backfill the gaps, providing girls with better ways to draw boundaries around social expectations of others and providing boys with better ways of expressing their feelings. Children are not responsible for the sexist systems and norms that they are born into, and our intention in this discussion is to animate how these norms appear to be operating in the lives of our participants without blaming the participants themselves.

A main theme that we identified related to navigating conflict in their social relationships, which is something that all participants experienced. For the boys, disengaging, removing themselves from the situation, and finding something else to do was a common strategy. Girls tended to engage with conflict through various means that revolved around their social relationships, whether that be relying on others for support or adjusting their behaviour and emotions to meet the needs of the relationship dynamic. This finding aligns with research by Brahnam and colleagues [65]. When investigating conflict resolution styles within the workplace, they identified that women tend to utilize a collaborative approach and men tend to avoid conflict outright. Based on our findings, it appears that these same trends emerge well before adulthood. Interestingly, while the boys isolated and the girls congregated, Alex, the non-binary participant in this study, appeared to intentionally engage in sociality for the purpose of reflection. They knew that to “recover” and approach the conflicts in their life they had to acknowledge who they were as a person (“I agree I was a weird kid”), focus on their internal mindset and view of themself (“I would try different self-esteem exercises”), and directly discuss the truth of the matter (“if something really bad happens then I don’t want to pretend like it never happened”). Growing up as non-binary and navigating cisnormative society, it appears that Alex resisted gendered norms by remaining reflexive and cognisant of themselves in the face of conflict. The world has forced them to come to terms with who they are—as it is “different” than what is expected and normalized—and this is reflected in their style of approaching conflict as well.

All study participants reported their friendships were of high importance, which we saw through both the quality and quantity of data relating to friendship. The boys in this study opted for friends who wanted to do the same things and participate in the same activities while the girls were drawn to friends who demonstrated similar traits, goals, and aspirations which inherently reinforced their own identity. This preference for those who are similar is not unexpected and appears in much of the literature related to youth social homophily [66–68]. While the rhetoric in society, and in Canadian classrooms especially [69–71], promotes inclusion and acceptance of difference, it appears that youth still place high salience on similarities. This desire for ‘sameness’ was also exposed in the ways that participants’ friends reinforced gendered expectations and performativity. For Nicholas this looked like excelling in academics to impress his boy peers and outperform his girl peers. Elizabeth had to ensure her appearance aligned with “the perfect girl” because her friends “all look the exact same because everything is this trend.”

Our study findings are in keeping with Kågesten and colleagues’ research (2016), which suggests that family are another central influence on the way that young people come to understand gender attitudes [72]. For example, the boys in our study were often influenced by their same-gender parent to “do more” and achieve academic excellence and the girls expressed worries about burdening their parents and the resultant behaviour change required to combat this fear. Perhaps what is most noteworthy about our findings is how well they align with past research, even research that is decades old; nothing is really new. Despite messaging that tells Canadian youth that we are moving toward gender equality and that everyone has the same opportunities available to them, gender inequality and gendered experiences still appear to strongly influence the ways that young people navigate social relationships. This is an important finding. Because mental health is so inherently tied to social relationships [14–18], the gendered dimensions of these relationships may be contributing to the gender gap in mental health experienced by Canadian young people, and that appears to disadvantage girls [8, 10–12].

Another new contribution that our findings make is related to non-binary young people. Alex, our one gender diverse participant, described how non-binary individuals had to enjoy “certain interests and hobbies” based on information they were receiving from their in-person and virtual social relationships. In the current literature base, one review highlights that young adolescents express stereotypical and inequitable gender attitudes and that these are often influenced by their peers [72]. While this review highlights that boys and girls experience differences in gender socialization processes, our study emphasizes that this pattern also appears to be occurring within the lives of gender diverse youth. Our findings suggest that gender diverse youth may be imposing constraints and reinforcing stereotypical non-binary norms in similar ways to cisgender young people, who are also reinforcing stereotypical gender norms in their social groups.

Overall, the incredible pressure of gendered norms, and the need to conform to norms to form gendered identity, echoes findings in a large body of academic literature (for example, Connell’s [73] seminal work on hegemonic masculinity). That this has not changed over decades should compel us to reconsider the kinds of education and supports that are available because they don’t appear to be helping young people with the problems they face. Take for example the boys in our study who removed themselves from conflict with their family members as a strategic mode of protecting their mental well-being. While we commend young men for enacting conflict resolution skills in the ways they know how, it places them at a disadvantage because they are not utilizing social support within the family dynamic. Evidence shows that parents are often a first line of defence in protecting against poor mental health for young people [28, 29] and thus if these relationships contain more unresolved conflict for boys, it places them at a higher risk because they are less likely to access familial support. While girls report being worse off in many mental health measures, boys do have higher rates of completed suicide [74, 75]. Perhaps one facet of this may be that boys are less likely to access family support.

When studies present a homogenous view of how young people from different genders approach relationships, the assumption that men are less able or less willing to participate in emotional and supportive relationships, and that women are uninterested in the sharing of mutual activities in their social connections, may be perpetuated [44]. Holding to this narrow view of gender and relationships leaves little room for a nuanced understanding of, or resistance to, the effect that gendered social norms hold on relationships, and in turn, mental health. It was notable that the girls in this study were especially attentive to the lack of emotional expression that is displayed by boys, with Ashley emphasizing that boys “have feelings and emotions” and Haley saying that it is “sad” that boys cannot display their full range of emotions. It gives one pause to wonder if this recognition and empathy for the boys in their lives add to the burden that girls are already experiencing. Both Sierra and Elizabeth described experiences of not wanting to burden their friends, families, or teachers. This is concerning and is in keeping with literature that indicates that those who require help (i.e. cancer patients, individuals with chronic pain) often report self-perceived burden [76], which in turn has negative implications for quality of life and mental health measures (i.e. depressive symptoms, suicidal ideation, anxiety, hopelessness) [76, 77]. Extrapolating these findings to the adolescent girls in our study who also exhibit self-perceived burden, it does not bode well for their mental health outcomes. The girl participants were the ones recognizing, resisting, and dismantling the stereotypical and gendered norms that exist in their boy peers’ lives, and not vice versa, which provides clues as to one potential reason that girls are disproportionately affected by the consequences of gender inequality. Yet something is also hopeful in these findings in that empathy can be a bridge to conflict and coping styles and can help people learn to read each other’s behaviour in more supportive and productive ways.

## Strengths and limitations

Our study had several strengths. First, the qualitative methodology within this investigation allowed for a deeper understanding of the nuances that exist within social relationships, and how gender influences young people in particular, to be explored. Additionally, the inclusion of youth as competent and valuable informants aligns with the UN Convention on the Rights of the Child [78] which emphasizes the rights of youth to be involved in matters that impact them. The youth who participated in our study were highly articulate and were generous in providing rich accounts of their own lives.

This study is not without limitations. The majority of our sample identified as cisgender and therefore the experiences of gender diverse youth are not as prominent. While we recognize that social relationships for young people who are gender diverse directly impact their mental health [31, 38, 79] and Alex displayed this to a certain degree within our study, further research with youth who are gender diverse and how they navigate social relationships would be an important next step. Additionally, other intersecting social identities such as race, socioeconomic status, and sexual orientation are known to relate to youth mental health [80–82] and these were not investigated closely in relation to our study topics. Finally, this study was conducted during the COVID-19 pandemic when social relationships were occurring in an atypical, primarily virtual, format. This likely influenced and contributed to the way that young people discussed their relationships.

## Conclusions

This study highlights the experiences of Canadian youth as they navigate their social relationships, and their impact on mental health. Social connections were reported as highly important to all participants. However, many gendered differences were identified. These were particularly salient in the ways that youth describe the interaction of gender and relationships; how youth manage conflict; and the dissatisfaction that girls express regarding gendered stereotypes, expectations, and norms.

Our findings highlight ways that youth can recognize and implement measures that protect and promote their mental health in their relationships. They suggest that bolstering young people’s ability to capitalize on their strengths, while also encouraging new modes of mental health promotion (e.g. filling stereotypically gendered gaps in coping skills) for all youth is important. For example, adults can work to provide boys with skills to manage and express their emotions and girls with strategies to develop boundaries or navigate feeling overburdened by others’ expectations. Additionally, adults must acknowledge the pressures non-binary youth experience and be intentional to provide more constructive support and guidance to these young people. This study supports the importance of adults working to model social relationships that aren’t subjected to the gendered norms that are perpetuating the narrative that girls/boys/gender diverse youth need to navigate their social relationships in a particular gendered way. Finally, at a structural level, our findings draw attention to the harmful messages that are telling girls they are a burden, and the need to dismantle these messages so that all young people have the opportunity to flourish.

Canadian policies claim to prioritize gender equality. Yet, the findings of this study emphasize that gender norms, stereotypes, and sexism are still prominent in the lives of young people. Clearly, current approaches to dismantle these limiting harmful gendered norms are not overly effective and all young people in Canada deserve better. This paper serves as a wakeup call to how little progress is being made to stop constraining gendered norms in the area of social relationships, which in turn shape mental health disparities. This paper is also a call to action to the adult duty bearers in society: to unpack this gender gap in robust and intentional ways that lead to tangible changes in how young people are socialized and taught to navigate relationships and conflict. Otherwise, there is little reason to think that the same patterns will not continue to persist in the lives of future generations.

## Supporting information

S1 File Semi-structured interview guide. (DOCX)

## References

[1] Canadian Mental Health Association. Fast Facts About Mental Health and Mental Illness [Internet]. Toronto (ON): Canadian Mental Health Association; 2021 July 19 [cited 2024 July 19]. Available from: https://cmha.ca/brochure/fast-facts-about-mental-illness/

[2] Patalay P, Demkowicz O. Debate: Don’t mind the gap-why do we not care about the gender gap in common mental health difficulties? Child and Adolescents Mental Health. 2023;28(2): 341–43. doi: 10.1111/camh.12647 37032492

[3] McIsaac MA, King N, Steeves V, Phillips SP, Vafaei A, Michaelson V, et al. Mechanisms accounting for gendered differences in mental health status among young Canadians: A novel quantitative analysis. Preventative Medicine. 2023;169. doi: 10.1016/j.ypmed.2023.107451 36796589

[4] McIsaac MA, Reaume M, Phillips SP, Michaelson V, Steeves V, Davison CM, et al. A novel application of a data mining technique to study intersections in the social determinants of mental health among young Canadians. SSM Popul Health. 2021;16. doi: 10.1016/j.ssmph.2021.100946 34746359 PMC8551646

[5] Connolly MD, Zervos MJ, Barone CJ, Johnson CC, Joseph CLM. The mental health of transgender youth: Advances in understanding. J Adolesc Health. 2016;59(5), 489–95. doi: 10.1016/j.jadohealth.2016.06.012 27544457

[6] Rosenfield S, Mouzon D. Gender and mental health. In: Aneshensel CS, Phelan JC, Bierman A, editors. Handbook of the sociology of mental health. Springer: Dordrecht; 2013. pp. 277–96. 10.1007/978-94-007-4276-5_14

[7] Scandurra C, Mezza F, Maldonato NM, Bottone M, Bochicchio V, Valerio P, et al. Health of non-binary and genderqueer people: A systematic review. Front Psychol. 2019;10(1453). doi: 10.3389/fpsyg.2019.01453 31293486 PMC6603217

[8] Campbell OLK, Bann D, Patalay P. The gender gap in adolescent mental health: a cross-national investigation of 566,829 adolescents across 73 countries. SSM Popul Health. 2021;13. doi: 10.1016/j.ssmph.2021.100742 33748389 PMC7960541

[9] Patalay P, Fitzsimons E. Development and predictors of mental ill-health and wellbeing from childhood to adolescence. Soc Psychiatry and Psychiatr Epidemiol. 2018;53:1311–1323. doi: 10.1007/s00127-018-1604-0 30259056

[10] Thomas JF, Temple JR, Perez R, Rupp R. Ethnic and gender disparities in needed adolescent mental health care. J Health Care Poor Underserved. 2011;22(1):101–10. doi: 10.1353/hpu.2011.0029 21317509 PMC3133675

[11] Van Droogenbroeck F, Spruyt B, Keppens G. Gender differences in mental health problems among adolescents and the role of social support: Results from the Belgian health interview surveys 2008 and 2013. BMC Psychiatry. 2018;18(1). doi: 10.1186/s12888-018-1591-4 29320999 PMC5763832

[12] Wiklund M, Malmgren-Olsson E-B, Öhman A, Bergström E, Fjellman-Wiklund A. Subjective health complaints in older adolescents are related to perceived stress, anxiety and gender-a cross-sectional school study in Northern Sweden. BMC Public Health. 2012;12(993). doi: 10.1186/1471-2458-12-993 23158724 PMC3533931

[13] Greene M, Patton G. Adolescence and gender equality in health. Journal Adolesc Health. 2020;66(1 Suppl): S1–S2. doi: 10.1016/j.jadohealth.2019.10.012 31866032 PMC6928587

[14] World Health Organization. Mental Health [Internet]. [unknown]: World Health Organization; 2022 June 17 [cited 2023 Dec 18]. Available from: https://www.who.int/news-room/fact-sheets/detail/mental-health-strengthening-our-response

[15] Mental Health Home [Internet]. Atlanta (GA): Centers for Disease Control and Prevention; 2024 Jan 25. Mental Health; [updated 2023 April 25; cited 2024 Jan 9]. Available from: https://www.cdc.gov/mentalhealth/learn/index.htm#:~:text=Mental%20health%20includes%20our%20emotional,others%2C%20and%20make%20healthy%20choices

[16] Health [Internet]. Ottawa (ON): Government of Canada; 2024 Mar 25. About Mental Health; 2020 June 22. [cited 2024 Jan 9]. Available from: https://www.canada.ca/en/public-health/services/about-mental-health.html

[17] Canadian Mental Health Association. The Importance of Human Connection [Internet]. Toronto (ON): Canadian Mental Health Association; 2019 Oct 17 [cited 2024 Jan 19]. Available from: https://cmha.ca/news/the-importance-of-human-connection/

[18] United Nations Human Rights Office of the High Commissioner. UN Expert highlights importance of social relationships for mental health and well-being [Internet]. Geneva: United Nations; 2019 June 24 [cited 2024 Jan 10]. Available from: https://www.ohchr.org/en/media-advisories/2019/06/un-expert-highlights-importance-social-relationships-mental-health-and

[19] Lamblin M, Murawski C, Whittle S, Fornito A. Social connectedness, mental health and the adolescent brain. Neurosci Biobehav Rev. 2017;80:57–68. doi: 10.1016/j.neubiorev.2017.05.010 28506925

[20] Capp G, Berkowitz R, Sullivan K, Astor R-A, De Pedro K, Gilreath TD, et al. Adult relationships in multiple contexts and associations with adolescent mental health. Res Soc Work Pract. 2016;26(6). doi: 10.1177/1049731515624967

[21] Moore GF, Cox R, Evans RE, Hallingberg B, Hawkins J, Littlecott HJ, et al. School, peer and family relationships and adolescent substance use, subjective wellbeing and mental health symptoms in Wales: a cross sectional study. Child Indic Res. 2018;11(6):1951–65. doi: 10.1007/s12187-017-9524-1 30524519 PMC6244918

[22] Holt-Lunstad J. Social connection as a public health issue: The evidence and a systemic framework for prioritizing the “social” in social determinants of health. Annu Rev Public Health. 2022;43:193–213. doi: 10.1146/annurev-publhealth-052020-110732 35021021

[23] Kok BE, Fredrickson BL. Wellbeing begins with “we”: The physical and mental health benefits of interventions that increase social closeness. In: Huppert FA, Cooper CL, editors. Interventions and Policies to Enhance Wellbeing: Wellbeing: A complete reference guide, Volume 4. John Wiley & Sons; 2013. doi: 10.1002/9781118539415.wbwell042

[24] Santini ZI, Koyanagi A, Tyrovolas S, Mason C, Haro JM. The association between social relationships and depression: A systematic review. J Affect Disord. 2015;175:53–65. doi: 10.1016/j.jad.2014.12.049 25594512

[25] Wang J, Mann F, Lloyd-Evans B, Ma R, Johnson S. Associations between loneliness and perceived social support and outcomes of mental health problems: A systematic review. BMC Psychiatry. 2018;18(156). doi: 10.1186/s12888-018-1736-5 29843662 PMC5975705

[26] Veenstra R, Laninga-Wijnen L. The prominence of peer interactions, relationships, and networks in adolescence and early adulthood. In Crockett LJ, Carlo G, Schulenberg JE, editors. APA handbook of adolescent and young adult development. American Psychological Association; 2023. 10.1037/0000298-014

[27] Blum RW, Lai J, Martinez M, Jessee C. Adolescent connectedness: cornerstone for health and wellbeing. Adolescent Wellbeing. 2022;379. doi: 10.1136/bmj-2021-069213 36302526 PMC9600165

[28] Hassett A, Green C, Zundel T. Parental involvement: A ground theory of the role of parents in adolescent help seeking for mental health problems. SAGE Open. 2018;8. doi: 10.1177/2158244018807786

[29] Rickwood DJ, Mazzer KR, Telford NR. Social influences on seeking help from mental health services, in-person and online, during adolescence and young adulthood. BMC Psychiatry. 2015;15(40). doi: 10.1186/s12888-015-0429-6 25886609 PMC4355380

[30] Foster CE, Horwitz A, Thomas A, Opperman K, Gipson P, Burnside A, et al. Connectedness to family, school, peers, and community in socially vulnerable adolescents. Child Youth Serv Rev. 2017;81:321–31. doi: 10.1016/j.childyouth.2017.08.011 30202142 PMC6128354

[31] Westwater JJ, Riley EA, Peterson GM. What about the family in youth gender diversity? A literature review. Int J Transgend. 2019;20(4): 351–70. doi: 10.1080/15532739.2019.1652130 32999622 PMC6913651

[32] Pulimeno M, Piscitelli P, Miani A, Colao A, Colazzo S. Training teachers as health promoters. Journal of Interdisciplinary Research Applied to Medicine. 2020;4(1):37–46. doi: 10.1285/i25327518v4i1p37

[33] Mazzer KR, Rickwood DJ. Community-based roles promoting youth mental health: comparing the roles of teachers and coaches in promotion, prevention and early intervention. Int J Mental Health Promot. 2013;15(1):29–42. doi: 10.1080/14623730.2013.781870

[34] Miller-Lewis LR, Sawyer ACP, Searle AK, Mittinty MN, Sawyer MG, Lynch JW. Student-teacher relationship trajectories and mental health problems in young children. BMC Psychology. 2014;27. doi: 10.1186/s40359-014-0027-2 25685350 PMC4317136

[35] Geulayov G, Borschmann R, Mansfield KL, Hawton K, Moran P, Fazel M. Utilization and acceptability of formal and informal support for adolescents following self-harm before and during the first COVID-19 lockdown: Results from a large-scale English schools survey. Front Psychiatry. 2022;13. doi: 10.3389/fpsyt.2022.881248 35815012 PMC9263724

[36] Boyd CP, Hayes L, Nurse S, Aisbett DL, Francis K, Newnham K, et al. Preferences and intention of rural adolescents toward seeking help for mental health problems. Rural Remote Health. 2011;11(1):1582. 21319934

[37] Roach A. Supportive peer relationships and mental health in adolescence: An integrative review. Issues Ment Health Nurs. 2018;39(9):723–37. doi: 10.1080/01612840.2018.1496498 30252560

[38] Cavaligos T., Anxiety depression, and peer support in transgender and gender diverse youth. Doctoral Dissertation, Pacific University. 2018.

[39] Kim HH-S. School context, friendship ties and adolescent mental health: A multilevel analysis of the Korean Youth Panel Survey (KYPS). Soc Sci Med. 2015;145:209–16. doi: 10.1016/j.socscimed.2015.05.002 25960374

[40] Jakobsen AL, Hansen CD, Andersen JH. The association between perceived social support in adolescence and positive mental health outcomes in early adulthood: a prospective cohort study. Scand J Public Health. 2022;50(3);404–11. doi: 10.1177/1403494821993718 33645305

[41] Landstedt E, Asplund K, Gådin KG. Understanding adolescent mental health: the influence of social processes, doing gender and gendered power relations. Sociol Health Illn. 2009;31(7):962–78. doi: 10.1111/j.1467-9566.2009.01170.x 19659740

[42] Hay DF, Nash A, Caplan M, Swartzentruber J, Ishikawa F, Vespo JE. The emergence of gender differences in physical aggression in the context of conflict between young peers. Br J Dev Psychol. 2011;29(Pt 2):158–75. doi: 10.1111/j.2044-835X.2011.02028.x 21592146

[43] Rose AJ, Rudolph KD. A review of sex differences in peer relationship processes: Potential trade-offs for the emotional and behavioral development of girls and boys. Psychol Bull. 2006;132(1):98–131. doi: 10.1037/0033-2909.132.1.98 16435959 PMC3160171

[44] McKenzie SK, Collings S, Jenkin G, River J. Masculinity, social connectedness, and mental health: Men’s diverse patterns of practice. Am J Mens Health. 2018;12(5):1247–61. doi: 10.1177/1557988318772732 29708008 PMC6142169

[45] Fiori KL, Denckla CA. Social support and mental health in middle-aged men and women: A multidimensional approach. J Aging Health. 2012;24(3):407–38. doi: 10.1177/0898264311425087 22396474

[46] Grav S, Hellzèn O, Romild U, Stordal E. Association between social support and depression in the general population: the HUNT study, a cross-sectional survey. J Clin Nursing. 2011;21(1–2):111–20. doi: 10.1111/j.1365-2702.2011.03868.x 22017561

[47] Weiss MR, Smith AL. Friendship quality in youth sport: Relationship to age, gender, and motivation variables. Journal of Sport & Exercise Psychology. 2002;24(4):420–37. doi: 10.1123/jsep.24.4.420

[48] Camara M, Bacigalupe G, Padilla P. The role of social support in adolescents: Are you helping me or stressing me out? Int J Adolesc Youth. 2017;22(2):123–36. doi: 10.1080/02673843.2013.875480

[49] Bokhorst CL, Sumter SR, Westenberg PM. Social support from parents, friends, classmates, and teachers in children and adolescents aged 9 to 18 years: Who is perceived as most supportive? Social Development. 2009;19(2):417–26. doi: 10.1111/j.1467-9507.2009.00540.x

[50] Rice SM, Purcell R, McGorry PD. Adolescent and young adult male mental health: Transforming system failures into proactive models of engagement. J Adolesc Health. 2018;62(3S):S9–S17. doi: 10.1016/j.jadohealth.2017.07.024 29455724

[51] Pattyn E, Verhaeghe M, Bracke P. The gender gap in mental health service use. Soc Psychiatry Psychiatr Epidemiol. 2015;50(7):1089–95. doi: 10.1007/s00127-015-1038-x 25788391

[52] Smyth N, Buckman JEJ, Naqvi SA, Aguirre E, Cardoso A, Pilling S, et al. Understanding differences in mental health service use by men: an intersectional analysis of routine data. Soc Psychiatry Psychiatr Epidemiol. 2022;57(10):2065–77. doi: 10.1007/s00127-022-02256-4 35318495 PMC9477949

[53] Brand E, Rodriguez-Monguio R, Volberg R. Gender differences in mental health and substance use disorders and related healthcare services utilization. Am J Addict. 2019;28(1):9–15. doi: 10.1111/ajad.12826 30536669

[54] Wadge S, Lethby M, Stobbe K, Gardner P, Michaelson V. Gender matters: Exploring the mental health of youth experiencing homelessness in Canada. International Journal on Homelessness. 2024;4(2):200–21. doi: 10.5206/ijoh.2023.3.16751

[55] DuPont-Reyes MJ, Villatoro AP, Phelan JC, Painter K, Link BG. Adolescent views of mental illness stigma: An intersectional lens. Am J Orthopsychiatry. 2019;90(2):201–11. doi: 10.1037/ort0000425 31380669 PMC7000296

[56] Latalova K, Kamaradova D, Prasko J. Perspectives on perceived stigma and self-stigma in adult male patients with depression. Neuropsychiatr Dis Treat. 2014;10:1399–405. doi: 10.2147/NDT.S54081 25114531 PMC4122562

[57] Lynch L, Long M, Moorhead A. Young men, help-seeking, and mental health services: Exploring barriers and solutions. Am J Mens Health. 2018;12(1):138–49. doi: 10.1177/1557988315619469 27365212 PMC5734535

[58] Dolotina B, Turban JL. A multipronged, evidence-based approach to improving mental health among transgender and gender-diverse youth. JAMA Netw Open. 2022;5(2). doi: 10.1001/jamanetworkopen.2022.0926 35212756

[59] Wittlin NM, Kuper LE, Olson KR. Mental health of transgender and gender diverse youth. Annu Rev Clin Psychol. 2023;19:207–32. doi: 10.1146/annurev-clinpsy-072220-020326 36608332 PMC9936952

[60] Sandelowski M. Focus on research methods: Whatever happened to qualitative description? Res Nurs Health. 2000;23(4):334–40. doi: 10.1002/1098-240x(200008)23:4&lt;334::aid-nur9&gt;3.0.co;2-g 10940958

[61] Sandelowski M. What’s in a name? Qualitative description revisited. Res Nurs Health. 2010;33(1):77–84. doi: 10.1002/nur.20362 20014004

[62] Braun V, Clark V. Thematic analysis: A practical guide. Sage; 2022.

[63] Ravitch SM, Carl NM. Qualitative research: Bridging the conceptual, theoretical, and methodological. 2nd ed. Sage; 2021.

[64] Vasileiou K, Barnett J, Thorpe S., Young T. Characterising and justifying sample size sufficiency in interview-based studies: systematic analysis of qualitative health research over a 15-year period. BMC Med Res Methodol. 2018;18: 1–8. doi: 10.1186/s12874-018-0594-7 30463515 PMC6249736

[65] Brahnam SD, Margavio TM, Hignite MA, Barrier TB, Chin JM. A gender-based categorization for conflict resolution. Journal of Management Development. 2004;24(3):197–208. doi: 10.1108/02621710510584026

[66] Laursen B. Making and keeping friends: The importance of being similar. Child Dev Perspect. 2017;11(4):283–89. doi: 10.1111/cdep.12246

[67] Richmond AD, Laursen B, Stattin H. Homophily in delinquent behavior: The rise and fall of friend similarity across adolescence. Int J Behav Dev. 2018;43(1). doi: 10.1177/0165025418767058

[68] Trinh SL, Lee J, Halpern CT, Moody J. Our buddies, ourselves: The role of sexual homophily in adolescent friendship networks. Child Dev. 2018;90(1):e132–e147. doi: 10.1111/cdev.13052 29574690 PMC6157003

[69] Galczynski M, Tsagkaraki V, Ghosh R. Unpacking multiculturalism in the classroom: Using current events to explore the politics of difference. Can Ethn Stud. 2012;43(3):145–64.

[70] Hymel S, Katz J. Designing classrooms for diversity: Fostering social inclusion. Educational Psychologist. 2019;54(4):331–9. doi: 10.1080/00461520.2019.1652098

[71] Lam M. Effects of Canada’s increasing linguistic and cultural diversity on educational policy, programming, and pedagogy. Journal of Contemporary Issues in Education. 2019;14(2). doi: 10.20355/jcie29370

[72] Kågesten A, Gibbs S, Blum RW, Moreau C, Chandra-Mouli V, Herbert A, et al. Understanding factors that shape gender attitudes in early adolescence globally: A mixed methods systematic review. PLoS one. 2016;11(6). doi: 10.1371/journal.pone.0157805 27341206 PMC4920358

[73] Connell RW. Gender and power: Society, the person, and sexual politics. Polity Press; 1987.

[74] Oliffe JL, Kelly MT, Montaner GG, Links PS, Kealy D, Ogrodniczuk JS. Segmenting or summing the parts? A scoping review of male suicide research in Canada. Can J Psychiatry. 2021;66(5):433–45. doi: 10.1177/07067437211000631 33719600 PMC8107953

[75] Public Health Agency of Canada. Suicide in Canada: Key Statistics (infographic) [Internet]. Ottawa (ON): Government of Canada; [modified 2023 Jan 9; cited 2024 Jan 9]. Available from: https://www.canada.ca/en/public-health/services/publications/healthy-living/suicide-canada-key-statistics-infographic.html

[76] Wilson KG, Curran D, McPherson CJ. A burden to others: A common source of distress for the terminally ill. Cogn Behav Ther. 2005;34(2):115–23. doi: 10.1080/16506070510008461 15986788

[77] Kowal J, Wilson KG, McWilliams LA, Péloquin K, Duong D. Self-perceived burden in chronic pain: Relevance, prevalence, and predictors. Pain. 2012;153(8):1735–41. doi: 10.1016/j.pain.2012.05.009 22703692 PMC3999031

[78] UN General Assembly. Convention on the Rights of the Child. [1989] Available from:https://www.ohchr.org/sites/default/files/Documents/ProfessionalInterest/crc.pdf

[79] Eisenberg ME, McMorris BJ, Rider GN, Gower AL, Coleman E. “It’s kind of hard to go to the doctor’s office if you’re hated there.” A call for gender-affirming care from transgender and gender diverse adolescents in the United States. Health Soc Care Community. 2020;28(3):1082–89. doi: 10.1111/hsc.12941 31917883 PMC7124990

[80] Gajaria A, Guzder J, Rasasingham R. What’s race got to do with it? A proposed framework to address racism’s impacts on child and adolescent mental health in Canada. J Can Acad Child Adolesc Psychiatry. 2021;30(2):131–7. doi: 10.1016/j.childyouth.2018.08.030 33953765 PMC8056965

[81] Reiss F. Socioeconomic inequalities and mental health problems in children and adolescents: A systematic review. Soc Sci Med. 2013;90:24–31. doi: 10.1016/j.socscimed.2013.04.026 23746605

[82] Hatzenbuehler ML. Advancing research on structural stigma and sexual orientation disparities in mental health among youth. J Clin Child Adolesc Psychol. 2017;46(3):463–75. doi: 10.1080/15374416.2016.1247360 27911583 PMC6759852

