# Supplementary material for: Navigating (gendered) social worlds: A qualitative exploration of Canadian young people’s social relationships and mental health
Source: PLOS Ment Health. 2024 Nov 13;1(6):e0000113. doi: 10.1371/journal.pmen.0000113 (PMC12798626; doi:10.1371/journal.pmen.0000113)
Supplement: S1 File — (DOCX) [file pmen.0000113.s001.docx]

**S1 File: Semi-structured interview guide for: Navigating (gendered) social worlds: A qualitative exploration of Canadian young people’s social relationships and mental health**

1. Opening question (purpose is to locate our study in participant’s everyday life overall)

What do you see as some of the day-to-day challenges and opportunities that you experience as a young person?

- Are you answering this question differently than you would have before the pandemic? Please explain.

1. Exploring Coping.

I want you to think about a time that was a struggle for you. Maybe it was super stressful, or you felt sad or depressed, angry or overwhelmed, or something else entirely. Can you tell me about it?

*Prompts to explore participant’s story*

- In that situation (or story) what kind of strategies did you use to cope?
- What kind of resources were available to you? Did you use them (why or why not?)
- I’d like to hear about the strengths that you brought to that situation. What were some of the challenges?
- Were there things that made you feel better able to help yourself, or able to get any help you needed? Please explain. What kind of things made you feel less empowered, or less able to get the outcomes that you wanted?
- Has your experience of being able to cope with things changed during the pandemic? Please explain.

1. Exploring intersectional expectations.

Do you think people you encounter in your daily life expect certain behaviours from you because of your gender? Can you give me some examples? What’s that like for you?

*Prompts to explore participant’s intersectional experiences*

- Can you tell me about the expectations that might restrict some of the things you do?
- Do you think these expectations are the same as or different from those that people from other ethnic groups experience.
- How about the expectations that might lead to opportunities?
- Where do you get the messages that tell you what the expectations are? (probe for parents, other adults, peers, media, etc.)
- How do you think these expectations relate to the story you told me at the beginning, about the time you had a struggle? Did the expectations effect the outcomes of that story/situation? Please explain.

1. Concluding questions

- From your own experiences, what advice would you give to someone your age who is struggling with mental health?
- What advice would you give to someone who was trying to support them? What kinds of things could they do or should they not do?
- Do you have any ideas about what you hope society, and the decisions makers in Canada, will learn about supporting mental health of young people so that we can do a better job moving forward?
- Is there anything else you would like to tell me?
